# Chronic Rhinotillexomania Leading to Unilateral External Nare Stenosis

**DOI:** 10.7759/cureus.3172

**Published:** 2018-08-21

**Authors:** Abhishek Gupta, Anurag Dhingra

**Affiliations:** 1 Geriatrics, Center for Addiction and Mental Health/University of Toronto, Toronto, CAN; 2 Family Medicine, Star Medical Center, Mississauga, CAN

**Keywords:** rhinotillexomania, nose-picking, body-focused repetitive behaviors, chronic rhinitis, nasal stenosis

## Abstract

Compulsive nose picking (rhinotillexomania) is a commonly known condition to general practitioners and pediatricians and is often advised against. This case highlights a 29-year-old individual with a prolonged history of intermittent rhinotillexomania who presented with repetitive viral upper respiratory infections. Physical examinations showed an enlarged nasal turbinate unilaterally with an otherwise normal nasal architecture. Further imaging and investigations showed no abnormalities or evidence of greater pathology. It was hypothesized that the patient's rhinotillexomania induced repetitive inflammation and subsequent hyperplasia of the nasal tissue, narrowing the circumference of an external nare. This case highlights the risks of a common but potentially dangerous habit along with its management.

## Introduction

Digital nasal exploration and hair removal is a benign activity often found at near-universal levels in the general population [1]. Significant portions of the population, primarily adolescents, have been found to engage in this activity up to four times a day [2]. Although taken for granted as an unattractive habit by the common population, it has severe implications, including nasal septum perforation, epistaxis, higher respiratory infection risk, and even underlying anxiety based psychiatric disorders [2-3]. When this otherwise benign habit develops to such degrees that it interferes with an individual's social interaction, daily functioning, or physical functions, it is linked to rhinotillexomania, a form of trichotillomania focused on the nasal area [1].

## Case presentation

Initial presentation

The patient is a 29-year-old male of Asian Indian origin, who presented for symptoms of a viral upper respiratory infection (URI). Initial symptoms included a clear nasal discharge for the past four days, mild nasal and sinus congestion, general myalgia, and a low-grade fever of 100.3°F. Further physical examination showed erythematous nasal turbinates with a distinct lack of cervical lymphadenopathy, tonsillar exudates, sinus tenderness, or pharyngeal erythema. As such, a clinical diagnosis of viral URI was made and treated accordingly with over-the-counter (OTC) acetaminophen-nasal decongestant formulations. However, a review of past medical history showed that the patient had a similar episode of viral URI approximately two months ago that was treated in a similar manner at a different outpatient clinic. The patient also disclosed performing nasal instrumentation along with daily, consistent contact with multiple family members who had similar URI symptoms prior to each personal episode of viral URI in the last six months. As the patient was solely concerned with the resolution of his URI at this first visit, the patient was discharged at his own request but volunteered to appear for a follow-up appointment in two weeks.

Subsequent follow-up visits involved a more extensive review of symptoms along with a more thorough nasal examination. The patient noted experiencing transient non-mucoid rhinorrhea in the morning on a near-daily basis for over two years. This rhinorrhea was usually very transient, lasting approximately one hour. His familial and personal medical history was insignificant for any conditions that may predispose to recurrent infections or any other pathology, especially those pertaining to an immune-compromised state. Specifically, he lacked any severe febrile symptoms or signs of sepsis. In addition, his history did not indicate any repetitive episodes of gastrointestinal or lower respiratory infections. Further, psychologically, he displayed an intact sensorium with no significant issues regarding his decision-making capacity, understanding, mood, or memory.

The patient described that he had started picking at coarse and thick nasal hair follicles that initially irritated his internal nasal cavity. Later, the patient began a prophylactic regimen of using metal instruments to selectively remove thicker hair follicles. This regimen had a paired compulsive aspect, often with a sense of anxiety and relief. Although it did not affect his daily functioning, the patient often felt irritation at not removing coarse nasal hair follicles and relief upon doing so. The patient maintained this habit for approximately one year, often with associated internal lacerations, temporary mucoid and bloody nasal discharge, local nasal tenderness, and inflammation. Subsequently, the patient observed that with every successive episode of inflammation, his left external nare underwent greater enlargement and stenosis. At a later stage, the patient noticed a reduced hair presence in his nasal cavity and local nasal tenderness, ultimately discouraging and reducing his regimen's frequency.

**Figure 1:**
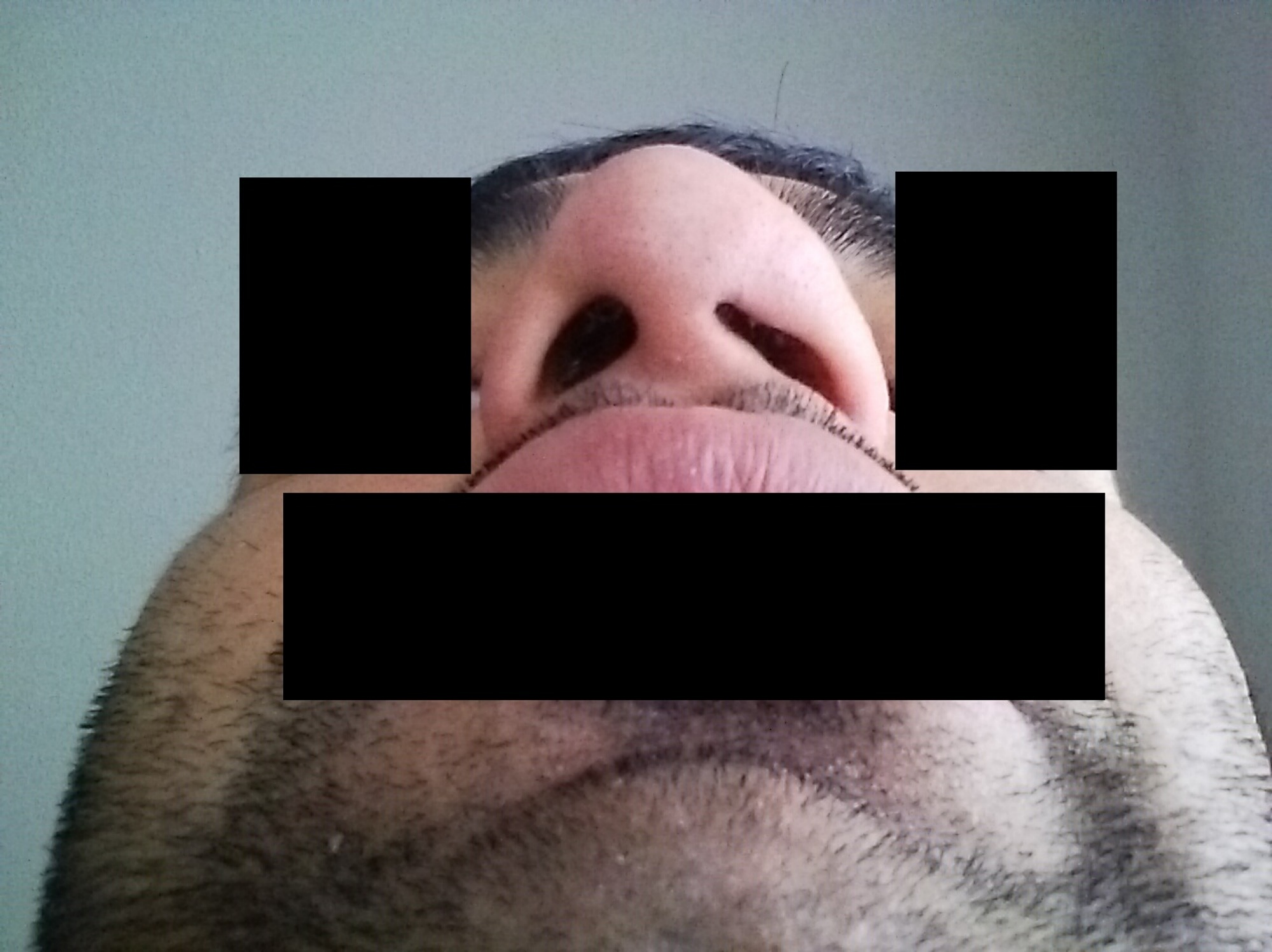
Overgrowth of the meatus blocks the left nasal opening

**Figure 2:**
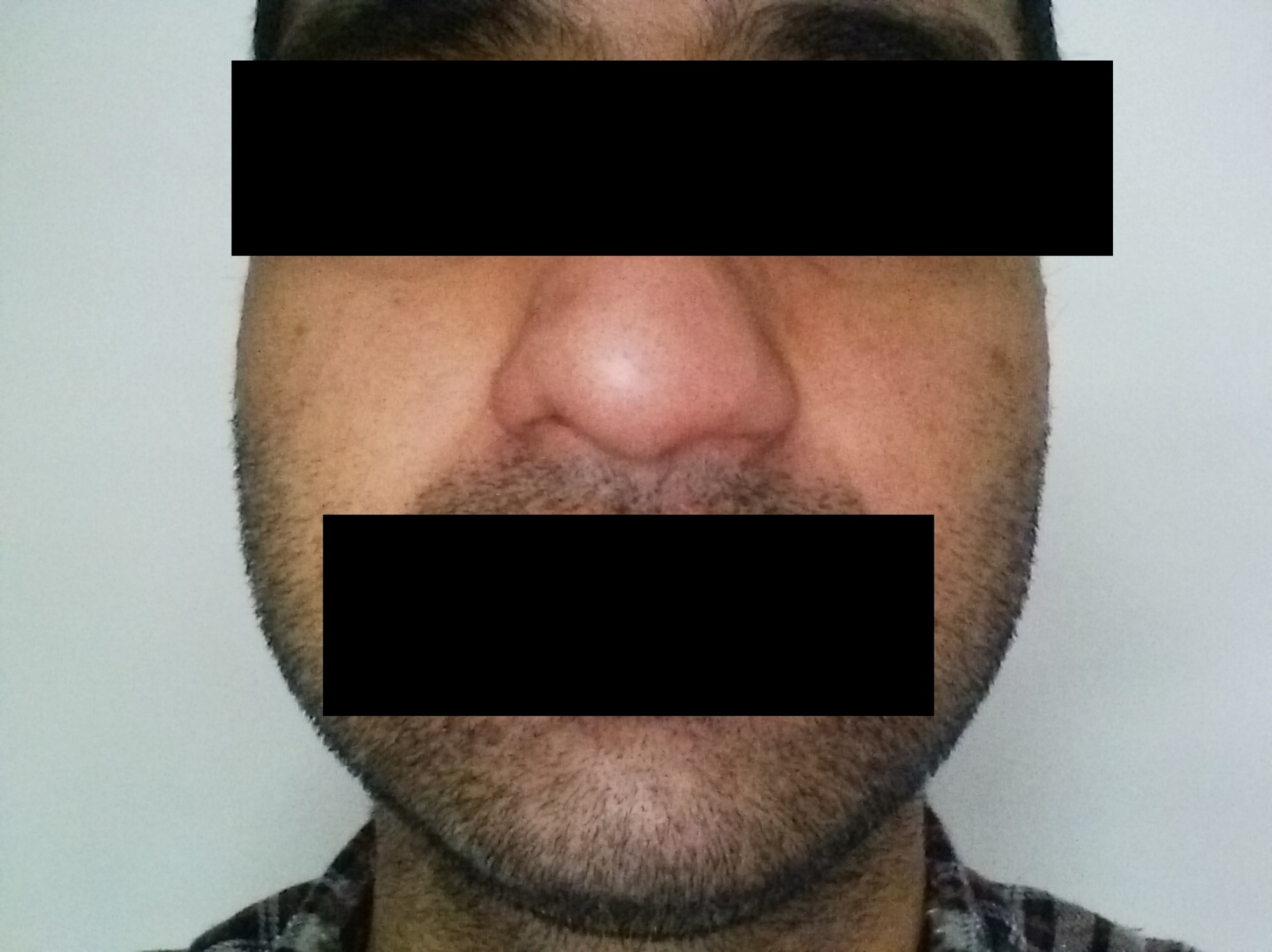
Abnormal growth of the left nare with unilateral loss of superficial nasal contours

On examination, the primary care team noticed that on passive breathing, both nares had sufficient and equal air flow. However, on forceful nasal expiration, the stenotic nostril had significantly reduced air flow compared to the intact nare. The patient's left nostril was significantly stenosed due to an expanded alar lobule, soft tissue facet, and a mild contralateral deviation of the columella (Figures 1-2). The nasal mucosa was erythematous and showed patchy lichenification. Computer tomography (CT) of the sinuses showed no evidence of alteration in the superior nasal and sinus cartilage beyond the external nare. His serum testing for metabolic or electrolyte abnormalities was insignificant. Otorhinolaryngology (ENT) consultation also confirmed the absence of any nasal polyps, septum perforation, or any other abnormality in the nasal tissue. A psychiatric evaluation identified this patient to have a generalized body-focused repetitive behavior (BFRB) disorder.

The Diagnostic and Statistical Manual of Mental Disorders (DSM-5) criteria for rhinotillexomania are based on the same criteria as trichotillomania but with a specific focus on the nasal mucosa. It states that hair picking should be in a pattern where it may or may not be noticeable (widely distributed or localized), with possible attempts to conceal/camouflage the hair loss, and the patient has made repeated attempts to stop or decrease the hair pulling. It is also important to the diagnosis that no other psychiatric or medical condition can be responsible for the hair loss [4].

Management

Given the already reduced scope of the nasal regimen, the lack of any significant respiratory impediment and the relatively greater side-effect potential of an antidepressant course, a trial of behavioral therapy was conducted. This regimen included regular nail trimming, continuous hand hygiene, and aversion conditioning (via malodorous stimuli on fingertips). Familial support was also encouraged in avoiding the same lifestyle changes. As part of his aversion therapy, the patient often dipped his second and third digits in malodorous oil. As such, any digital proximity to the nasal area produced a strong aversion response to his nasal habit. Regular digital nail trimming with sufficient hand hygiene also reduced the risk of any intranasal lacerations and its resulting infection. Finally, as the stenosis was cosmetic in nature, the patient declined any need for surgical correction. The prognosis was also improved based on the ego-dystonic nature of this case, where the patient already recognized the abnormal pattern as unhealthy. This motivated the patient to make significant attempts to correct this unhealthy behavior. Overall, while the physical nare stenosis was not reversed, the client reported a nearly complete cessation of both morning rhinorrhea episodes as well as nasal digital exploration episodes at the three-month interval of behavioral modification therapy.

## Discussion

Due to repetitive nasal trauma secondary to rhinotillexomania, the resulting infections led to a chronically inflamed state of this patient's nasal cartilage. It is theorized that this chronic inflammation led to adaptive hyperplasia of the same tissue, creating a stenosis of his nasal cavity. His symptom profile, although benign in appearance, had significant underlying medical associations.

Wertheim et al. [3] showed that the nose picking behavior was significantly associated with a higher carriage of Staphylococcus aureus (S. aureus) in the nasal area. This habit damages the nasal mucosa and creates traumatic lesions, exposing extracellular matrix molecules, such as fibronectin and collagen, and thus providing ideal colonization sites for S. Aureus. In addition, this colonization further exacerbates pruritus and irritation in the nasal area, which, in turn, further exacerbates the habit [3]. In the case discussed here, not only was the patient indulging in nose picking but also in repetitively removing hair follicles in the same area. As such, it could have exposed the extracellular matrix to a further degree, leading to even more severe traumatic responses. Therefore, the patient's rhinotillexomania greatly exposed the nasal tissue to infectious agents, likely increasing the risk of recurrent chronic sinusitis.

Further, the patient's symptom profile can be indicative of an underlying anxiety spectrum disorder [4]. His behavior originated as a cosmetic step but two elements in his regimen had significant psychiatric implications. At first, the patient imagined his coarse nasal hair as a bodily defect, removing them prematurely despite painful consequences, including prolonged nasal tenderness. Secondly, he derived a sense of relief from the act of removing nasal hair while its avoidance created anxiety. Therefore, both of these factors, the compulsion and the relief, have a strong resonance with an obsessive-compulsive pathology indicative of anxiety-based disorders such as BFRB [4]. Multiple cases have also shown an association between rhinotillexomania and generalized anxiety disorder (GAD), obsessive-compulsive disorder (OCD), or attention-deficit hyperactivity disorder (ADHD) [5-7]. Similarly, the medical consequences of rhinotillexomania include intranasal tissue erosion, recurrent epistaxis, and URIs [5-7].

## Conclusions

Rhinotillexomania, a BFRB disorder, has multiple associations with infectious and psychiatric risks. Such cases can present with a variety of symptoms, including recurrent epistaxis, upper respiratory infections, and abnormalities in the nasal cartilage tissue. This patient presented with a significant cosmetic stenosis of a nasal cavity along with recurrent URIs secondary to chronic rhinotillexomania. This case was managed with a thorough examination of the nasal tissue for anatomic defects as well as an extensive interview regarding the contributing behavior. Treatment options for rhinotillexomania focus on immediate symptomatic correction along with addressing the underlying psychiatric pathology. Standard treatment options for BFRB disorders include antidepressants and behavioral modification therapy. BFRB disorders should also warrant a thorough psychiatric evaluation for anxiety-spectrum psychiatric disorders (such as GAD or OCD) as well as ADHD.

## References

[1] Jefferson JW, Thompson TD (1995). Rhinotillexomania: psychiatric disorder or habit?. J Clin Psychiatry.

[2] Andrade C, Srihari BS (2001). A preliminary survey of rhinotillexomania in an adolescent sample. J Clin Psychiatry.

[3] Wertheim HF, van Kleef M, Vos MC, Ott A, Verbrugh HA, Fokkens W (2006). Nose picking and nasal carriage of Staphylococcus aureus. Infect Control Hosp Epidemiol.

[4] Grant JE, Stein DJ (2014). Body-focused repetitive behavior disorders in ICD-11. Rev Bras Psiquiatr.

[5] Rather YH, Sheikh AA, Sufi AR, Qureshi AA, Wani ZA, Shaukat TS (2011). ADHD presenting as recurrent epistaxis: a case report. Child Adolesc Psychiatry Ment Health.

[6] Giger R, Nisa L (2016). Demolition site: rhinotillexomania. Am J Med.

[7] Rathore D, Ahmed SK, Ahluwalia HS, Mehta P (2013). Rhinotillexomania: a rare cause of medial orbital wall erosion. Ophthalmic Plast Reconstr Surg.

